# Conformal Design of a High-Performance Antenna for Energy-Autonomous UWB Communication

**DOI:** 10.3390/s21175939

**Published:** 2021-09-03

**Authors:** Shobit Agarwal, Diego Masotti, Symeon Nikolaou, Alessandra Costanzo

**Affiliations:** 1 Department of Electrical, Electronic, and Information Engineering, Università di Bologna, 40136 Bologna, Italy; shobit.agarwal2@unibo.it (S.A.); alessandra.costanzo@unibo.it (A.C.); 2 Frederick Research Center, Frederick University, 1036 Nicosia, Cyprus; s.nikolaou@frederick.ac.cy

**Keywords:** UHF antennas, ultra wideband antennas, conformal antennas, radio frequency identification

## Abstract

In view of the need for communication with distributed sensors/items, this paper presents the design of a single-port antenna with dual-mode operation, representing the front-end of a future generation tag acting as a position sensor, with identification and energy harvesting capabilities. An Archimedean spiral covers the lower European Ultra-Wideband (UWB) frequency range for communication/localization purposes, whereas a non-standard dipole operates in the Ultra High Frequency (UHF) band to wirelessly receive the energy. The versatility of the antenna is guaranteed by the inclusion of a High Impedance Surface (HIS) back layer, which is responsible for the low-profile stack-up and the insensitivity to the background material. A conformal design, supported by 3D-printing technology, is pursued to check the versatility of the proposed architecture in view of any application involving its deformation and tracking/powering operations.

## 1. Introduction

With the rapid increase of so-called “smart dust” in the framework of the Internet of Things, i.e., small devices distributed in the environment able to perform basic sensing operations, there is an increased interest in zero-power pervasive computing [1]. In some cases, this operation can be performed by using inductive or resonant coupling [2,3]. However, this technique is applicable when there is a short distance between the charger and the device, and mainly static objects are involved. When these limitations need to be removed, i.e., moving objects far from the radiofrequency (RF) source have to be charged (such as in logistic applications), Energy Harvesting (EH) and/or Wireless Power Transfer (WPT) are the candidate technologies to make this feasible, because they can extend the battery lifetime or completely avoid its use. This can be done, for instance, by adopting Ultra High Frequency (UHF) combined with Radio Frequency Identification (RFID) technology, if the identification of the device is of primary importance important. Moreover, if we refer to applications with moving objects, operation as identification and localization both become, at the same time, of strategic importance: typically these functionalities are offered by separate wireless systems. The purpose of this paper is to present an advanced antenna solution representing the front-end of a future generation RFID tag able to effectively combine all the aforementioned needs, hence, to be an autonomous position sensor. For these purposes, the UHF band is exploited with the twofold goal of having a tag compatible with the existing RFID tag generations and being energy autonomous by means of the exploitation of dedicated RF energy showers [4]. As per the localization and communication operations, the antenna resort to the well-established Impulse Radio Ultra Wide Band (IR-UWB) technology, which has already demonstrated its effectiveness especially in indoor environment, by reaching sub-meter localization precision through the signal backscattered by the tag itself despite the ultralow power signals involved [4,5,6].

The proposed solution takes its inspiration from [7], where the authors designed a hybrid UWB-UHF antenna that has a good matching response, but with unsatisfactory performance from the axial ratio point of view. Further, designing an antenna which is insensitive to the background material is of paramount importance in case of the envisaged application as a tag of future generation. This makes an antenna that can be used wherever needed without changing the response according to the background material. For these reasons, the present work starts from the preliminary results of [8] where a first idea to shield the antenna was proposed, but for the UWB band only. Here, the shielding effect is extended for both the bands and a prototype realization is presented. To the authors’ knowledge, this is the first HIS able to guarantee both the circular polarization and low back radiation in a frequency band (in the literature just one of the two results is achieved at a time) while also acting as a shield at a completely different frequency (the UHF, in this case). The layout is then slightly changed because of conformal realization of the antenna: this additional step is made to test the versatility of the proposed architecture for future envisaged applications where a deformation is mandatory. As a final step, a planar balun is also added in order to guarantee a safe transition from the balanced antenna to the future diplexing network in microstrip technology responsible for the management of simultaneous UWB communication and UHF energy harvesting.

This paper is organized as follows. Related work is presented in Section 2. Section 3 discusses the design of circularly polarized hybrid antenna followed by the HIS structure design presented in Section 4. Further, modeling and fabrication of the proposed hybrid antenna with a suspended HIS structure and 3-D printed posts is presented in Section 5. Later, a dual-sided conformal design is discussed in Section 6 followed by the complete antenna structure with the inclusion of a balun for impedance transformation in Section 7. The article concludes in Section 8.

## 2. Related Work

The effective use of IR-UWB technology for the localization and tracking of moving items was first theoretically proved almost one decade ago [9,10], and more recently experimentally demonstrated [5,6,11]. For this reason, many solutions of new tags exploiting the UWB backscattering mechanism have been proposed in the recent past. Many of these solutions envisage the coexistence of both UWB and UHF standards, mostly for compatibility reasons, with the already existing RFID tags operating in the UHF band.

To cite some of them, in [3], a planar antenna is presented for dual-mode passive tag systems. The presented antenna operates in both UHF-RFID band and in the UWB band. A typical UHF-RFID antenna is designed on the top side of the substrate and the slot-loaded antenna for UWB communication is placed on the opposite side. The designed antenna can thus be used for item identification in the two bands and for precise tracking in indoor environments. In [12], a semi-passive UWB RFID system is designed. The tag consists of an RFID antenna operating at 2.4 GHz and a UWB antenna. The 2.4 GHz antenna is used, in this case, to wake up the tag, thus enhancing the battery lifetime. Similarly to [12], in [13] two co-located antennas are used for the dual-mode operation, but an 868 MHz signal is adopted for both waking up the tag and for ensuring compatibility with UHF RFID systems. Another hybrid UHF-UWB tag with two antennas on the same substrate is proposed in [14], where the dual-frequency behavior is just for compatibility with existing UHF RFID tags, not for energy harvesting purposes.

In addition, ref. [15] describes a planar hybrid UHF/UWB single antenna whose main body is used as an antenna at UHF to receive energy from a standard RFID reader, whereas a slot antenna obtained from the previous one, but with a separate port, transmits the UWB signal for communication. However, maintaining a low mutual coupling between the two ports was indeed a tough challenge. A planar inverted-F antenna (UHF band) with an ellipse shaped dipole (UWB band) is reported in [16] that has only identification and localization capabilities. To improve the isolation between the two ports, the design includes a two-port layout with inductive loading, and a complex differential feeding solution in the UWB band. A quarter elliptical patch along with two U-shaped striplines are used as antennas, but only for indoor positioning purposes in [17]: the proposed solution is compact, but it is incapable of mitigating high back radiation.

However, all the aforementioned designs require multi-port excitation and just a few of them are energy-aware, even if not battery-less, solutions. As an additional drawback, they do not support circular polarization (CP) in the UWB band. From the latter point of view, the literature offers CP solutions, as in [18,19], by they still are two-ports layouts with no energy autonomy functionality. In [18] the spiral UWB antenna uses two 100 Ω-RF resistors to achieve the needed axial ratio, and a planar loop loaded by meandered lines achieves CP also in the UHF band. The CP of the dual-band reader antenna in [19] is achieved by varying the shape of different patches acting as electric and magnetic dipoles in the UWB band, and through a complex feeding network piloting four suspended F-antennas in the UHF band.

Finally, in [7,20], the authors designed the first single-port hybrid UWB-UHF RFID antenna system for both energy harvesting and localization applications with backward compatibility with existing UHF RFID systems. A two-armed dipole antenna is used in the UHF band, and a typical CP UWB Archimedean spiral antenna is designed for localization and tracking purposes. A diplexer is also placed behind the spiral to guarantee high isolation between the two operating frequency bands. As a limitation, the designed antenna provides strong back radiation because of the absence of the ground plane, thus resulting strongly dependent on the background material where it lies on. The present paper tries to bridge this gap, with an improved and advanced antenna layout, also taking a look at the effect of a conformal design on the radiation performance. To the best of our knowledge, there are no solutions of dual-mode antennas in the UHF/UWB bands encompassing all the cited functionalities, i.e., high-performance dual-band operation that can be easily equipped with an energy harvesting unit, single-port layout, insensitivity to the back-ground material, and robustness with respect to deformation.

## 3. Antenna Design

**Figure 1:**
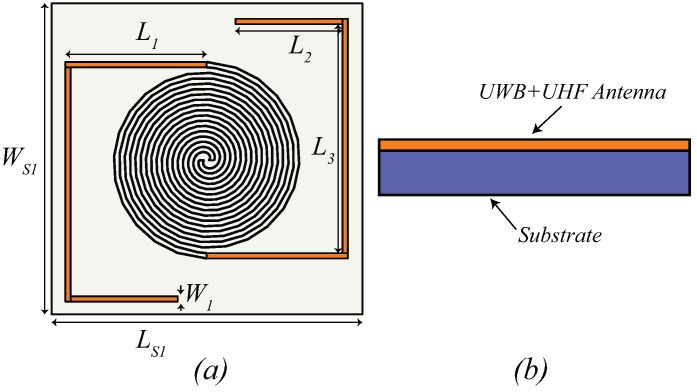
Iteration 1 design (**a**) Top view (**b**) side view.

A schematic representation of the antenna, referred to as Iteration 1 design in this article, with all the parameters is shown in Figure 1, and the optimized values of the design parameters are listed in Table 1. A paper substrate ($L_{S1}\times W_{S1}$) having dielectric constant, $\epsilon_{r}$ = 2.85, thickness, *h* = 5 mm, and tan δ = 0.053 is first used to design the antenna. From Figure 1, it can be observed that the presented structure is composed of two different antennas. An archimedean spiral antenna that operates in the lower European UWB frequency range (3.1–4.8 GHz) and responsible for communication/localization purposes. Meanwhile, the long dipole antenna, made of the meandered traces of the spiral plus the straight extensions (highlighted in orange in Figure 1a) antenna is operating at UHF band (868 MHz) and also considered as a receiving antenna to receive the electromagnetic (EM) energy able to guarantee its energy autonomy. Regarding the UWB antenna, the design rules follow the standard auto-complementary antenna rules [21], i.e., the copper and empty traces share the same width. The width and the number of turns have been optimized to reach an AR superior than in [7]. An increment in the width of the spiral and of the number of turns was recorded. Regarding the UHF antenna, its physical length is the result of the straight lines and of the spiral loops. In terms of radiation, the close loops cancel each other and the resulting far-field turns out to be horizontally polarized, as the main longer branches prolong the spiral arms. Moreover, the length of the dipole is greater than the standard half-wavelength, i.e., it is 2.5λ long, as can be evinced by the multiple resonances of the reflection coefficient plot.

**Figure 2:**
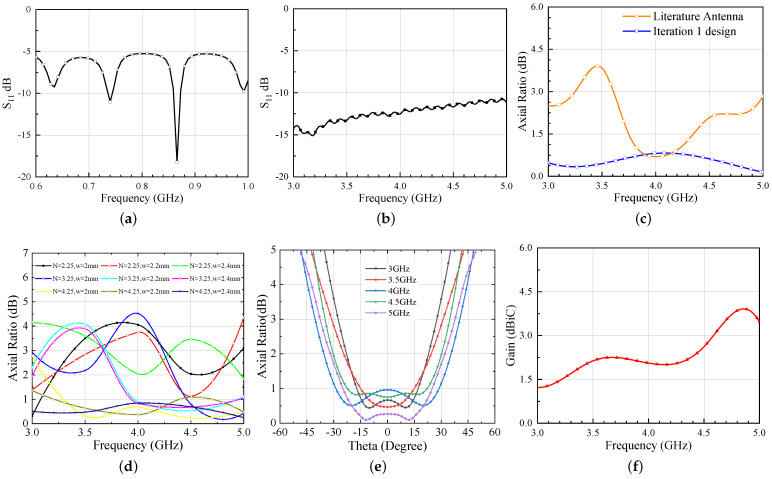
Simulated responses of hybrid UWB antenna (**a**) $S_{11}$ for UHF band (normalized to 12 Ω) (**b**) $S_{11}$ for UWB band (normalized to 120 Ω) (**c**) Axial Ratio v/s Freq (**d**) parametric study of AR optimization (**e**) Axial Ratio v/s Theta (**f**) Gain.

The simulated results of the Iteration 1 design are shown in Figure 2.

It is evident from Figure 2a,b that the antenna is operating well within the UHF and UWB frequency bands. The dipole antenna bandwidth (considering |$S_{11}$| at the antenna balanced port ≤−10 dB) is evaluated with respect to a 12 Ω resistance, because a rectifier, whose input resistance is typically in the range 10–15 ohm at low power levels, is foreseen as a load in this frequency band. Conversely, the UWB bandwidth is computed with respect to the standard impedance of an Archimedean spiral (120 Ω) [21]. In a spiral antenna, the critical parameters that contribute the most to achieve the circular polarization are the number of turns (N) and the width (w) of the traces [22]. Considering this, an extensive parametric study on the design presented in [7] is performed to achieve an improved circular polarization and the optimized response is shown in Figure 2c. Additionally, from Figure 2d, it can be seen that the most effective parameter is N and that a wider trace (hence a larger antenna footprint) is beneficial for the axial ratio, which is below 1 dB within the UWB range for N = 4.25 and w = 2.4 mm. Regarding the dipole length, it can be obviously tuned according to the desired matching condition. In the present application, the antenna length corresponds to the resonant condition (i.e., imaginary part of the antenna input port impedance equal to zero), but of course a proper tuning of the length can be exploited for conjugate matching condition fulfilment with the needed RFID chip impedance [7]. Additionally, the variation of AR w.r.t. to theta is shown in Figure 2e to gain an insight of its variation within the beamwidth. Figure 2f, demonstrates the gain performance of the Iteration 1 antenna. One can observe from the figure that the gain of the antenna slightly increases with the frequency.

**Figure 3:**
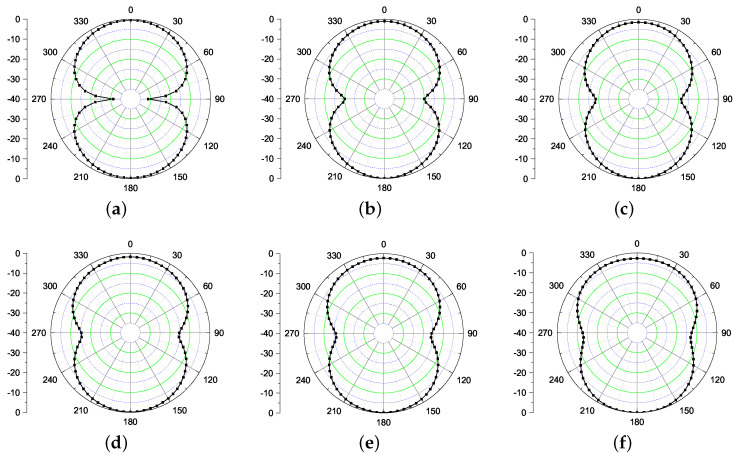
Simulated radiation patterns of Iteration 1 design (xz plane) at (**a**) 868 MHz (**b**) 3 GHz (**c**) 3.5 GHz (**d**) 4 GHz (**e**) 4.5 GHz (**f**) 5 GHz.

Next, the radiation performance of the antenna is studied as shown in Figure 3. The performance is shown at UHF frequency (868 MHz) along with frequencies in UWB range (3–5 GHz). It is evident from the results that the antenna radiates in θ = 0 ° and θ = 180 ° directions. This happens due to the fact that the structure is not backed by the ground plane that deteriorates the radiation properties of this antenna. Next, a plausible solution to mitigate the back radiation using a high impedance surface is discussed.

## 4. Design of High Impedance Surface

**Figure 4:**
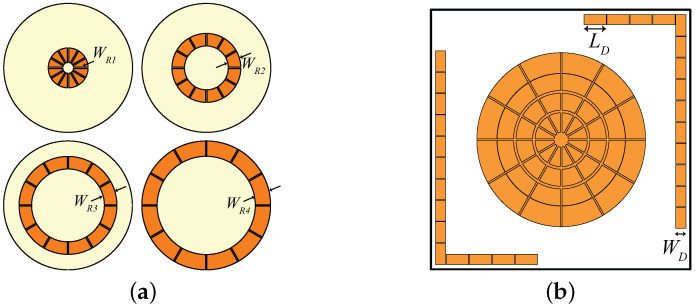
(**a**) Four HIS rings (innermost to outermost) and (**b**) complete HIS structure.

A high impedance surface (HIS), in general, is a combination of periodically arranged metallic patches placed over a grounded dielectric substrate [23]. HIS can be exploited to serve as a Perfect Magnetic Conductor (PMC). Unlike PEC, a PMC does not introduce any phase shift to the reflected waves; i.e., the incident and reflected wave remain coherent. The HIS structure for UWB spiral antenna designed on a 1-mm-thick paper substrate has already been reported in [8] (similar to the structure shown in Figure 4a). However, due to fabrication hindrance of achieving 1 mm paper thickness precision, the HIS is re-optimized on the Rogers/RT duroid 6002 ($\varepsilon_{r}$ = 2.94, tan*δ* = 0.0012, h = 0.508 mm) substrate. Furthermore, a novel inverted-L shaped HIS (to mitigate back radiation of UHF dipole) is also designed on the same substrate. A basic difference between rectangular and circular HIS is observed in the simulation setup. In rectangular HIS, the simulation can be performed using a unit cell. Only for the circular HIS must a unit ring be considered. A schematic of the complete layer and circular HIS is shown in Figure 4a,b, respectively. The optimized parameters of the HIS layer are summarized in Table 2 where $g_{r}$ and $g_{w}$ are the distance between the rings and gap between the patches within a ring, respectively. The response of the circular HIS is obtained in accordance with [24].

**Figure 5:**
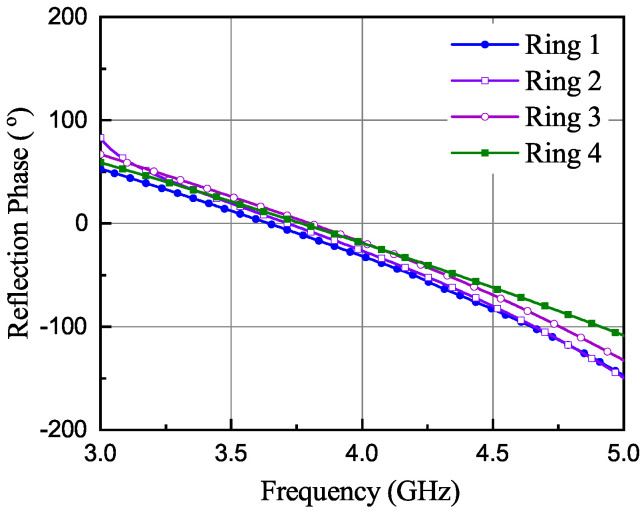
Phase response of four circular HIS rings.

More number of HIS rings can be can be utilized for achieving wideband performance. For this purpose, all the rings must have a similar phase response within the frequency band of interest [25]. In this work, four such rings are designed (Figure 4a) and their phase response is optimized for the UWB band. The optimized phase response of the four unit rings is shown in Figure 5 having a linear phase variation within the operating frequency range; then, in the following simulations, the HIS is considered as a whole (no more as a combination of four separated unit rings). A symmetric structure is maintained along both planes by introducing same number of patches, which in turn helps in improving circular polarization in UWB band. The surface phase response can be tuned within the desired frequency band by changing mostly the width ($W_{R1}$ – $W_{R4}$) in Figure 4. With increasing value of the widths, the phase response of Figure 5 shift towards lower frequency ranges. Also, the response shifts a bit towards right with increasing gap value between the patches. Moreover, the number of cells also plays an important role to have a linear phase response. This can be explained with mitigation of the curvature effect with increasing numbers of the patches within a ring.

Further, as shown in Figure 4b, a conventional inverted-L shaped HIS is designed [26], optimized, and inserted behind the dipole antenna operating at UHF band just to act as a shield, since linearly (horizontally in this case) polarized field is radiated at this frequency. Although the back radiation at the UHF range is not completely cancelled with L-shaped HIS (as can be seen in the next Figure 9a), it enhances the efficiency to 76% from 57% at the UHF band. Therefore, it can be concluded that HIS is playing a vital role to maintain a low profile stack up with satisfactory performance for both spiral and dipole antennas.

## 5. Antenna with Suspended HIS and 3-D Printed Posts

**Figure 6:**
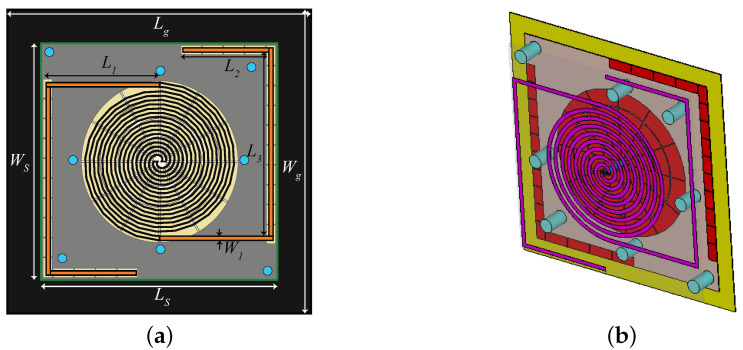
Antenna with suspended HIS and 3D printed posts: (**a**) front view; (**b**) perspective view (with transparent antenna substrate for ease of visualization). (Iteration 2 design).

As mentioned earlier, due to fabrications perplexities of thick paper substrate, the iteration 1 antenna is also re-designed on the Rogers/RO 6002 substrate with 1.5 mm thickness. Since the parameters of the substrate material are changed, the antenna need to be re-tuned for the desired performance. A schematic of the antenna (referred as Iteration 2 design in the article) is shown in Figure 6a,b, the top view and the perspective view, respectively. The corresponding parameters value of the antenna and the HIS are summarized in Table 3.

**Figure 7:**
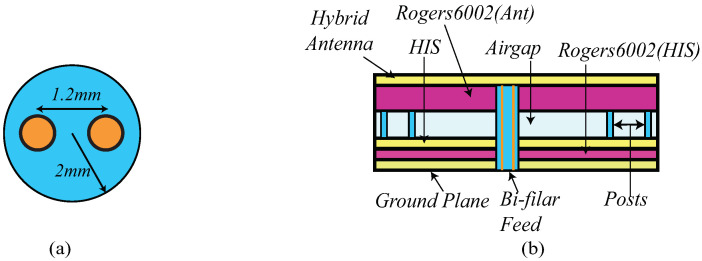
Bi-filar feeding (**a**) top view (**b**) Cross section view with antenna (not to scale).

A cross section view of the antenna is shown in Figure 7b. From the figure, it can be observed that Iteration 2 design comprises 6-layers viz. hybrid antenna, substrate, suspended air gap, HIS layer, substrate, and the ground plane. To enhance the gain performance of the antenna, which was previously affected by the lower substrate height, an air gap of 13 mm is introduced between the antenna and the HIS layer. To support the top layer, eight 3-D printed posts are utilized. The supporting posts (in light blue in the figure) are designed using 3D printing technology and PLA filament having $\varepsilon_{r}$ = 3.5 and tanδ = 0.04. The radius of the post, $R_{post}$, is 4 mm.

Furthermore, a novel feeding mechanism to feed the hybrid antenna is proposed in this work. A schematic of this feeding structure is shown in Figure 7a. It can be observed from the figure that one balanced bi-filar line is inserted within a PLA cylinder with radius of 2 mm. The distance between both lines is 1.2 mm that ensures a characteristics impedance of 120 Ω which is in accordance with standard impedance of the spiral antenna. The PLA cylinder serves two purposes: serving as the dielectric material for the bi-filar lines, and providing central support to the top layer (as shown in Figure 7b).

**Figure 8:**
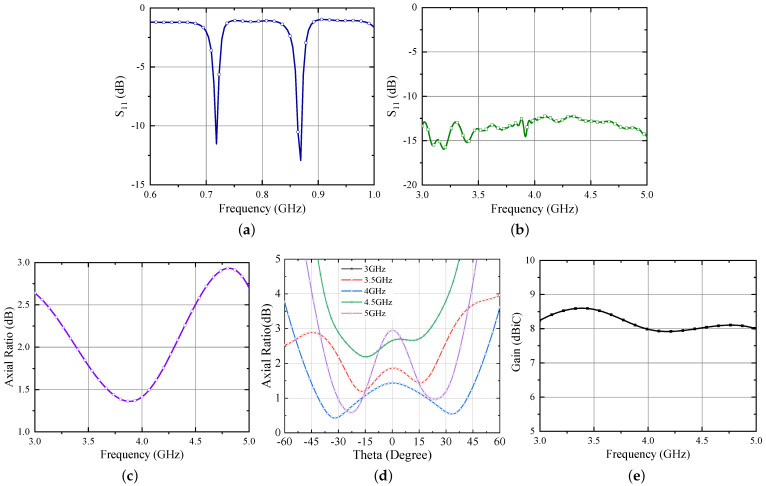
Simulated responses of hybrid UWB antenna (**a**) $S_{11}$ for UHF band (normalized to 12 Ω) (**b**) $S_{11}$ for UWB band (normalized to 120 Ω) (**c**) Axial Ratio v/s Freq (**d**) Axial Ratio v/s Theta (**e**) Gain.

The simulated results of the Iteration 2 design are shown in Figure 8. Similar to the previous design, the UHF antenna bandwidth is calculated assuming resistive part of a voltage-doubler rectifier for low incident power which is 12 Ω in this case and is displayed in Figure 8a. On the other hand, the UWB bandwidth is estimated with standard spiral antenna impedance [21] and shown in Figure 8b. From the results, it is evident that both antennas are operating well within the intended frequency band. However, a diplexing network would serve as a key element which can separate these two signals in the final realization of the next generation RFID tag. The design of this network will follow the rules already established in [7] and will be a future research activity.

Moving next, the axial ratio and gain results of Iteration 2 design are shown in Figure 8c–e, respectively. From Figure 8c,d, one can observe that the axial ratio of the antenna is below 3 dB which ensures circular polarization within the entire UWB range. Further, the suspended configuration improves the gain performance of the antenna. The simulated gain is ≥8 dBi for the UWB range and ∼4.8 dBi for the UHF band.

**Figure 9:**
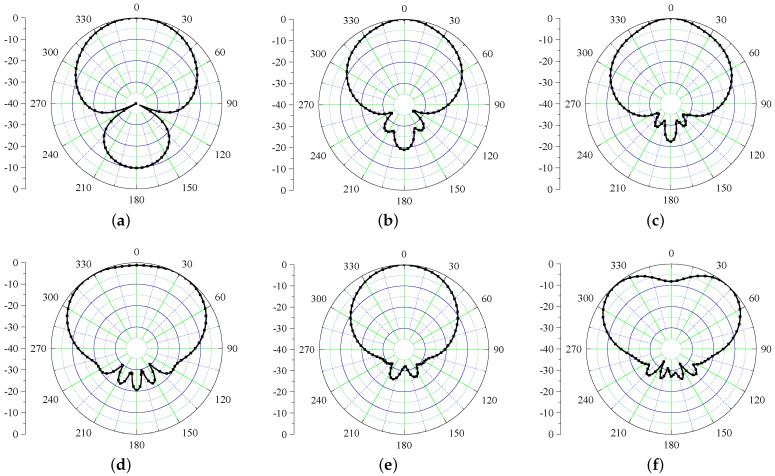
Simulated radiation patterns of Iteration 2 design (xz plane) at (**a**) 868 MHz (**b**) 3 GHz (**c**) 3.5 GHz (**d**) 4 GHz (**e**) 4.5 GHz (**f**) 5 GHz.

Since the HIS is serving as a PMC, the radiation characteristics of the Iteration 2 design are expected to be improved. The simulated E-field radiation patterns of the antenna are shown in Figure 9 at six different frequencies covering UHF and UWB frequencies. It is evident from the figures that after inclusion of HIS, the back radiation is improved by 10 dB for UHF band and more than 20 dB for the UWB band.

### Fabrication and Measurement

**Figure 10:**
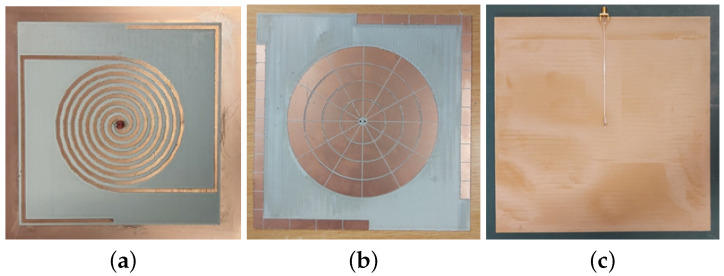
Fabricated prototype (**a**) Hybrid antenna layer (**b**) HIS layer (**c**) Ground back FR-4 layer with feed line.

To prove the veracity of the design, a prototype of the Iteration 2 design is fabricated and reflection coefficient characteristics were measured using Keysight PNA Network Analyzer E8368B (Keysight Technologies, Santa Rosa, CA, USA). The fabricated prototype is shown in Figure 10.

**Figure 11:**
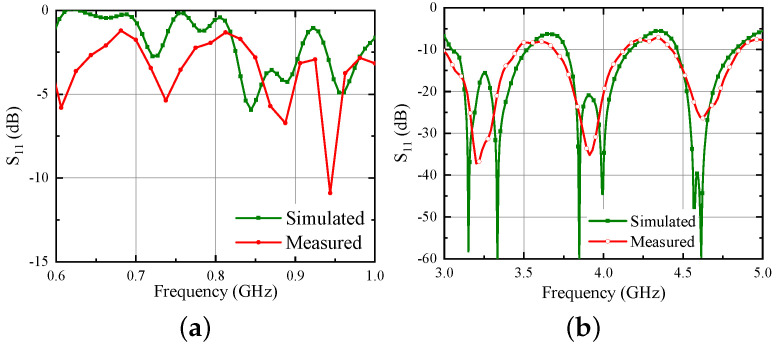
Simulated and measured reflection coefficient of the hybrid UWB antenna (**a**) $S_{11}$ for UHF band (normalized to 120 Ω) (**b**) $S_{11}$ for UWB band (normalized to 120 Ω).

Considering the difficulty to measure the response directly using balanced bi-filar lines by directly attaching an instrument, one of the two conductors of the bi-filar line is connected to a 120 Ω microstrip line (Figure 10c). To facilitate this, a FR4 substrate ($\varepsilon_{r}$ = 4.4, *h* = 1.6 mm) layer is added behind the ground plane to host the microstrip line and to provide support to the SMA connector used to excite the 120 Ω feed line. The other conductor of the bi-filar line is connected to the ground plane. This abrupt transition has been realized just to have a first measurement capable of demonstrating the correctness of the complex design at this stage. Of course, the correct solution to this issue would be the realization of a planar balun able also to transform the impedance level from 120 Ω to 50 Ω. This additional step has been performed later and is presented in Section 7. Unfortunately, the corresponding fabrication was not possible in reduced time because of the pandemic situation. The simulated and experimental results (both normalized to 120 Ω) are shown in Figure 11. From the figure, it can be seen that the results are in quite good agreement;of course, they are different from the earlier presented results because of the unconventional transition from bi-filar to microstrip line adopted in the measurement.

## 6. Dual Side Conformal Antenna

**Figure 12:**
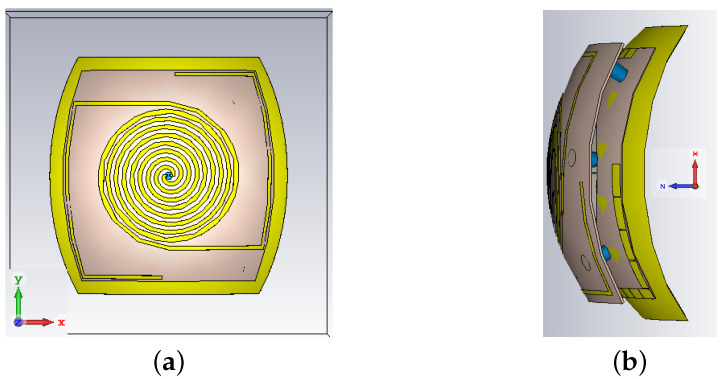
Conformal antenna design (**a**) Top view (**b**) side view.

In this section, a conformal design of the suspended antenna is discussed. Despite the usage of rigid substrates, this investigation is motivated by the fact that a future realization of the antenna (with the diplexer, the energy harvesting circuitry and the UWB backscatter modulator) is foreseen with flexible materials in order to be easily located in a wide range of placements and for a wide selection of applications where localization/tracking and energy autonomy are needed. One envisaged possibility is on the fuselage of a drone for charging it wirelessly while flying. All the parameters of this antenna are identical to the Iteration 2 design discussed in the previous section. A schematic of the conformal antenna is shown in Figure 12. As shown in the figure, the bending is performed around a hemisphere with a radius of 100 mm.

**Figure 13:**
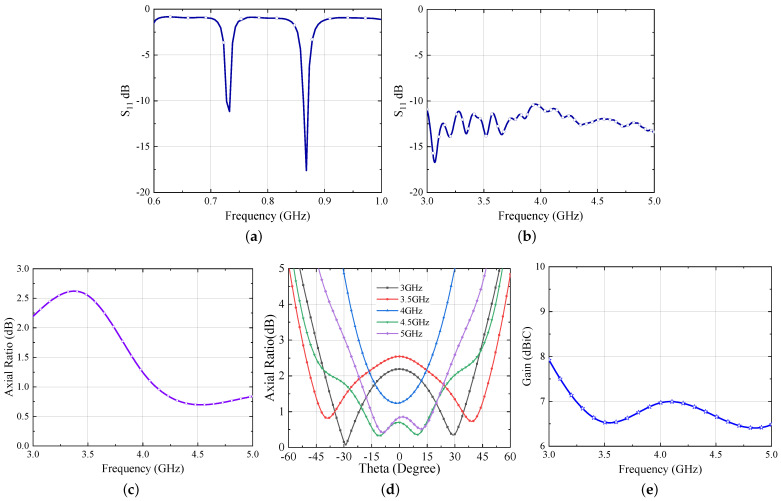
Simulated responses of hybrid UWB antenna (**a**) $S_{11}$ for UHF band (normalized to 12 Ω) (**b**) $S_{11}$ for UWB band (normalized to 120 Ω) (**c**) Axial Ratio v/s Freq (**d**) Axial Ratio v/s Theta (**e**) Gain.

Here one thing to note is that, during bending of the stacked antenna, it was observed that the inverted-L shaped HIS was no more behind the UHF antenna as expected. As a consequence of this, the effect of the HIS at the UHF frequency was completely lost. To mitigate this problem, the size of the HIS layer is scaled up by a factor of 1.05 which results in the total size of 126 × 126 mm^2^. The simulated responses of the conformal antenna are shown in Figure 13a–e.

One can observe from the results that the hybrid antenna is operating both in the UHF and UWB band, as expected. Moreover, the axial ratio performance of the antenna is still intact and it provides circular polarization for the entire UWB range despite the significant bending of the antenna layer. Figure 13e displays the gain of the conformal antenna. By comparing this with the Iteration 2 design, the gain of the antenna reduces because of the bending; however, it is still high enough for the communication purposes. As mentioned earlier, after bending, the UHF antenna becomes more vulnerable as it is present at the edges of the substrate; hence, a significant reduction is observed in the gain at UHF band and is 1 dBi at 868 MHz.

**Figure 14:**
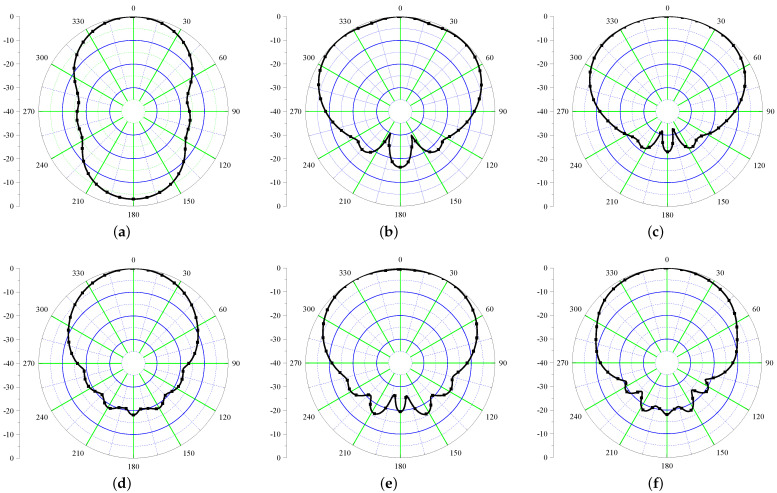
Simulated radiation patterns of conformal antenna (xz plane) at (**a**) 868 MHz (**b**) 3 GHz (**c**) 3.5 GHz (**d**) 4 GHz (**e**) 4.5 GHz (**f**) 5 GHz.

Moving further, the simulated radiation characteristics of the conformal antenna at UHF and within UWB frequency range are shown in Figure 14. Here, the major back radiation is observed at the UHF band (a front-to-back ratio of 3 dB is observed). This is due to the fact that, as the antenna is bent in both the planes, the UHF antenna which is already at the edge of the substrate becomes vulnerable to the back radiation. It is worth noticing that this performance is achieved thanks to the slight increase of the HIS previously described. At UWB frequencies (Figure 14b–f), the back radiation is below −15 dB.

## 7. Antenna with Balun

**Figure 15:**
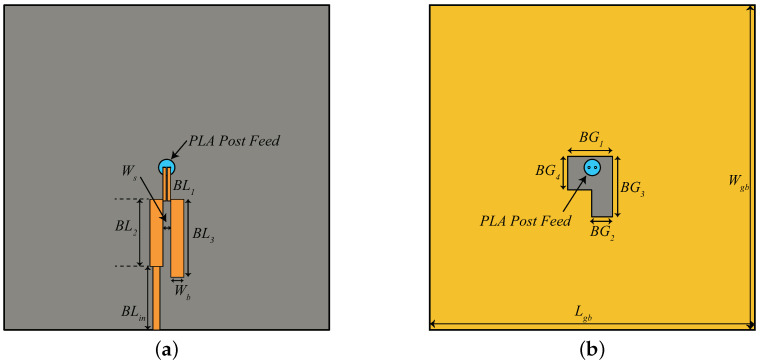
Balanced to Unbalanced (Balun) design (**a**) Top view (**b**) ground plane.

As discussed earlier, to feed the spiral antenna using the bifilar line feeding structure reported in this article, a balun is a strategic part to be developed for a direct connection of the dual-mode antenna to a 50 ohm instrumentation. One such balun is designed according to the rules given in [27] on a 0.508-mm-thick Rogers/RO 6002 substrate ($\varepsilon_{r}$ = 2.94, tanδ = 0.0012) as shown in Figure 15. It consists of an un-grounded planar 120 Ω bi-filar line, as a prosecution of the vertical one whose top side is the port 1, followed by an open stub (partially grounded) and a microstrip line (grounded) plus a microstrip impedance step to reach the 50 Ω at the output port (port 2). The parameters of the balun are summarized in Table 4.

**Figure 16:**
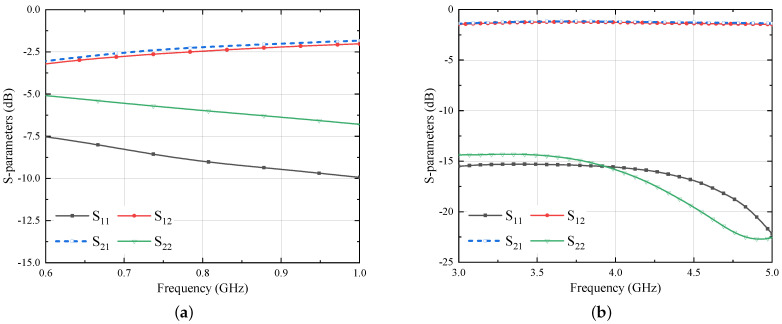
Simulated S-parameter results of planar balun at (**a**) UHF band (**b**) UWB band.

The simulated results of the balun structure shown in Figure 15 are displayed in Figure 16. As shown in Figure 16a, the transmission parameter between port 1 and 2 varies between −1.2 dB and −1.5 dB within the UWB band. Slightly worse, but still acceptable, performance are achieved at 868 MHz. Of course, the balun design is the result of a delicate trade-off between the performance in the two distant frequency ranges. Next, this balun is deployed behind the Iteration 2 design. One can note that the removal of the ground plane has been limited as much as possible in order not to affect the role of the HIS, even if this cannot be completely assured, as described later.

**Figure 17:**
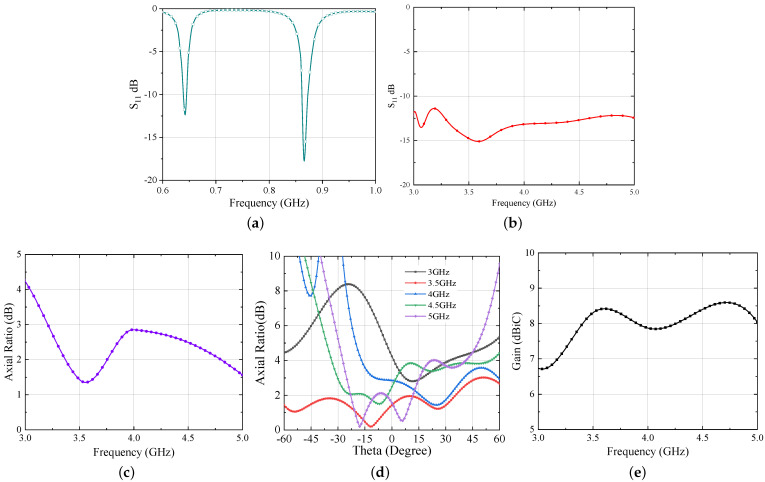
Simulated responses of hybrid UWB antenna (**a**) $S_{11}$ for UHF band (normalized to 12 Ω) (**b**) $S_{11}$ for UWB band (normalized to 120 Ω) (**c**) Axial Ratio v/s Freq (**d**) Axial Ratio v/s Theta (**e**) Gain.

The schematic of the balun inserted at the bottom side of Iteration 2 design is similar to Figure 15. However, due to the large ground plane of the Iteration 2 antenna (140 mm × 140 mm), the value of 50 Ω feed line length ($BL_{in}$) is increased to 52.6 mm. In addition, the ground plane and the substrate size is increased to 140 mm × 140 mm. The simulated results of the antenna integrated with the balun are shown in Figure 17.

From the figures, it can be observed that the antenna is operating well within the frequency bands of interest. However, the axial ratio of the antenna is compromised a little at the beginning of the UWB band: it remains below 3 dB from 3.22 GHz and provides circular polarization up to 5 GHz. The gain of the antenna is also good with a peak value of 8.6 dBi at 4.7 GHz with an average gain of more than 7 dBi in the entire UWB band, and 3 dBi at the UHF frequency. The improved gain performance of the present multi-mode antenna with respect to previous realizations, lead the authors to state that 10 m of distance from the reader/source for both tracking/localization [5] and energy transmission [28] in the UWB and UHF bands, respectively, could be exceeded. This will be part of our future research activity.

**Figure 18:**
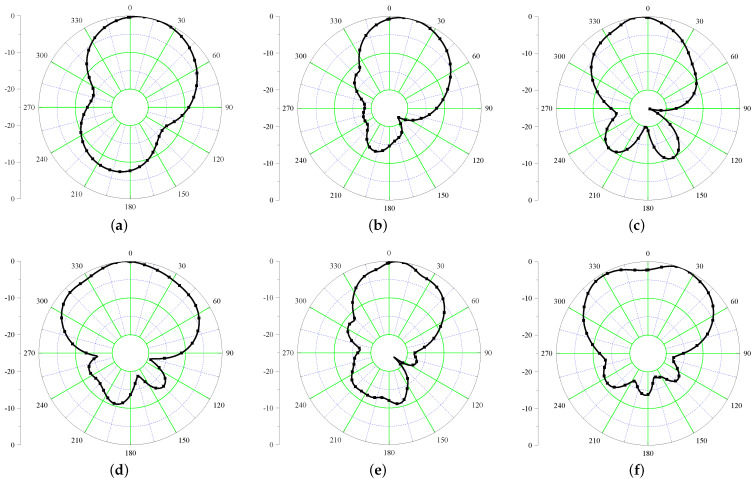
Simulated radiation patterns of conformal antenna (xz plane) at (**a**) 868 MHz (**b**) 3 GHz (**c**) 3.5 GHz (**d**) 4 GHz (**e**) 4.5 GHz (**f**) 5 GHz.

Next, the radiation characteristics of the antenna are computed at six different frequencies as shown in Figure 18.

From the figures, it can be concluded that after insertion of the Balun, the performance of the antenna is intact and is able to mitigate the back radiation at UHF as well as UWB band. However, it can be seen that after insertion of the balun the radiation patterns are slightly slanted. This happened due to the asymmetric removal of the ground plane behind the HIS to ascertain the balun performance. It is also worth noting that, because of the compact layout of the balun, its behavior, not shown for the sake of brevity, is not significantly affected while bending the whole system.

A comparative study of the proposed antenna was carried out with the existing literature and the results are summarized in Table 5. From the table, it is evident that the main advantages of the proposed antenna are its single-port nature that guarantees an easier feeding network, as well as a more compact layout, the circular polarization within the UWB band, and its platform independent behavior thanks to the HIS layer. Further, the robustness of the proposed antenna with respect to dual side bending, not always studied in the literature, is quite encouraging. Therefore, from the presented data, it can be concluded that the proposed antenna represents a step forward if compared with the existing literature designs.

## 8. Conclusions

A circularly polarized hybrid conformal antenna serving dual purposes of energy autonomy and UWB communication is presented in this article. A suspended high impedance surface is designed and deployed behind the antenna offering, for the first time, a simultaneous control of the back-radiation mechanism at highly separated frequencies, and of the circular polarization in the UWB band. The exploitation of 3D-printed posts plays a strategic role for both the mechanical stability and the original bi-filar feeding strategy of the proposed multilayer architecture. Furthermore, a conformal design of the proposed hybrid antenna is studied for future technological systems, drones for example, that would require wireless charging while flying: despite the bidirectional bending, the structure reveals itself to be quite robust to mechanical changes. The proposed radiating system will be able to play a crucial role as an RFID tag of the next generation as soon as it is equipped with the diplexing network, the harvesting section, and the back-scatter modulator. 

## Figures and Tables

**Table 1:** Parameters of Iteration 1 design.

Parameter	Value (mm)
$L_{s1}$	130.20
$W_{s1}$	130.20
$L_{1}$	63.60
$W_{1}$	2.40
$L_{2}$	48.20
$L_{3}$	103.00

**Table 2:** Optimized parameters of HIS layer.

Parameter	Value (mm)
$W_{R1}$	10.10
$W_{R2}$	9.49
$W_{R3}$	9.42
$W_{R4}$	9.49
$g_{r}$	0.6
$g_{w}$	0.4
$W_{D}$	5.5
$L_{D}$	9.8

**Table 3:** Optimized Parameters of Iteration 2 design and HIS.

Parameter	Value (mm)	Parameter	Value (mm)	Parameter	Value (mm)	Parameter	Value (mm)
$W_{S}$	120	$L_{1}$	58.4	$W_{R1}$	10.10	$g_{r}$	0.6
$W_{g}$	140	$L_{2}$	52.2	$W_{R2}$	9.49	$g_{w}$	0.4
$L_{S}$	120	$L_{3}$	94.6	$W_{R3}$	9.42	$W_{D}$	5.5
$L_{g}$	140	$W_{1}$	2.2	$W_{R4}$	9.49	$L_{D}$	9.8

**Table 4:** Optimized dimensions of the proposed metasurface.

Parameter	Value (mm)	Parameter	Value (mm)	Parameter	Value (mm)	Parameter	Value (mm)
$BL_{1}$	5	$BG_{1}$	20	$BL_{in}$	8	$L_{gb}$	60
$BL_{2}$	12.4	$BG_{2}$	9.3	$W_{b}$	2.4	$W_{gb}$	60
$BL_{3}$	14.4	$BG_{3}$	27	$BG_{4}$	15	$W_{s}$	1.4

**Table 5:** Comparison of the proposed antenna with the existing literature.

Ref.	Separate Antennas	No. of Antenna Ports	Circular Polarization (in UWB Band)	Insensitive to Background Material	Robustness wrt Deformation	Energy Harvesting Arrangement
[3]	yes	2	no	no	N.A.	no
[7]	no	1	yes	no	N.A.	yes
[15]	no	2	no	no	N.A.	yes
[16]	yes	2	no	yes	N.A.	no
[17]	yes	2	no	no	N.A.	no
[18]	yes	2	yes	yes	N.A.	no
[19]	yes	2	yes	yes	N.A.	no
This work	no	1	yes	yes	yes	yes

## Data Availability

Not applicable.
